# Reliability and Validity of the Indonesian Version of the McCloskey/Mueller Satisfaction Scale

**DOI:** 10.1155/2023/9999650

**Published:** 2023-07-13

**Authors:** I. Gede Juanamasta, Yupin Aungsuroch, Mary L. Fisher, Nursalam

**Affiliations:** ^1^Faculty of Nursing, Chulalongkorn University Bangkok, Bangkok, Thailand; ^2^Nursing Program, STIKes Wira Medika Bali, Bali, Indonesia; ^3^Indiana University School of Nursing, Indianapolis, IN, USA; ^4^College of Nursing, University of Florida, Gainesville, FL, USA; ^5^Faculty of Nursing, Universitas Airlangga Mulyorejo, Surabaya, East Java 60115, Indonesia

## Abstract

**Aim:**

To examine the reliability and validity of the Indonesian translation of the McCloskey/Mueller Satisfaction Scale.

**Background:**

The McCloskey/Mueller Satisfaction Scale is an instrument to measure nurses' feelings about their job. Although the instrument has existed for a long time, there has been a lack of studies using this questionnaire in Indonesia due to the limitation of instrument validation in the Indonesian version. Therefore, it is necessary to investigate the validation and reliability of the instrument.

**Methods:**

The instrument was validated with a cross-sectional study of 350 nurses. For purposes of transcultural adaptation and psychometric validation, a forward-back translation strategy was used in conjunction with an expert panel and a cross-sectional study. The validity of the construct was verified using confirmatory factor analysis, and the overall fit of the model was measured using the calculated fit indices. The standard deviation of the extracted variance was used to evaluate the convergence validity. Composite reliability and Cronbach's alpha coefficients were used to investigate the internal consistency of the study.

**Results:**

According to the results of the reverse translation, the Indonesian and original instruments are statistically indistinguishable. As a result of the confirmatory factor analysis, it was determined that the Indonesian version of the McCloskey/Mueller Satisfaction Scale adhered closely to the original structure of the English version. The convergence validity of the construct (0.44–0.74), reliability (Cronbach *α* = 0.69–0.93), and internal consistency (0.49–0.79) were rated highly.

**Conclusions:**

Good homogeneity and construct validity have been shown for the Indonesian translation of the McCloskey/Mueller Satisfaction Scale in the context of healthcare practice and research. *Implications for Nursing Management.* Nursing policymakers in Indonesia can use the instrument to inform the development of practice policies aimed at improving job satisfaction among nurses in the country.

## 1. Introduction

Nurse satisfaction is an important factor in nursing quality and turnover intentions [1, 2]. Despite being studied for a long time, the importance of individuals' feelings for their job is increasing. There are several nurse job satisfaction instruments based on motivational theories [3], which include the Job Satisfaction Survey by Spector [4], and the Job Descriptive Index (JDI) and the Job in General Scale (JIG) by Smith [5]. Others use human need theory [6], including Index Work Satisfaction (IWS) by Stamps [7] and the National Database of Nursing Quality Indicators (NDNQI)–adapted IWS by Taunton [8]. The McCloskey/Mueller Satisfaction Scale (MMSS) was developed based on the combined human need theory and motivational theory [3]. Thus, MMSS would give different perspectives compared to the job satisfaction instruments commonly used for nurses.

### 1.1. Background

The McCloskey/Mueller Satisfaction Scale (MMSS) is a comprehensive survey instrument developed for registered nurses working in hospitals [9]. For the first time, the instrument was developed with 36 distinct items. Mueller then reviewed the instrument to make it valid, reliable, and easy to use. Three items were excluded, and two items did not connect with any other dimensions, so that was the reason MMSS has now become 31 items [9].

Conceptually, MMSS was constructed by eight dimensions, including satisfaction with external awards (3 items), satisfaction with service schedules (6 items), satisfaction with the balance between family and work (3 items), satisfaction with relationships with coworkers (2 items), satisfaction with the opportunity to interact in the work environment (4 items), satisfaction with the opportunity to develop professionally (4 items), satisfaction with public recognition (4 items), and satisfaction with responsibility (5 items) [9]. It is related that when McCloskey-Mueller developed this instrument, they divided hospital rewards into three categories that included safety, social, and psychological. Satisfaction with maternity leave support, childcare facilities, direct supervisor support, relationships with peers, and opportunities to mingle with coworkers were all seen to be part of the social dimension of job satisfaction. Work responsibilities, educational possibilities, positive feedback, and a sense of agency in one's work were all factors in the minds of employees when it came to their jobs [9].

The former study reviewed factors related to nurse job satisfaction measured by MMSS [2]. The instrument has been widely used in many settings, including tertiary hospital, acute care hospital, mental health hospital, community, nursing homes, home healthcare, and urban and rural areas. Although it was designed for nurses, other studies have also applied it to evaluate the satisfaction of other professionals, such as case managers, midwives, and physicians. Another finding from Al-Qahtani et al. [2] showed that work environment, emotional state, social support, career ladder, and accountability had a significant impact on nurse satisfaction.

In a previous study [10], five experts in nursing administration calculated the content validity index for the Indonesian version of MMSS (I-MMSS), and CVI ranged from 0.92 to 1. Pearson's correlation with a correlation greater than 0.4 established the validity of the criterion. In addition, convergent validity was used with an average variance extracted (AVE) greater than 0.54. The total scale's Cronbach's alpha coefficient was 0.86, and the composite reliability was 0.89. However, the previous study did not measure this instrument comprehensively, and specifically, the translation process was not discussed, nor were construct validity and composite reliability results presented. This study has measured the construct validity and reliability of MMSS based on its psychometric properties.

A previous psychometric study from the USA revealed that MMSS was valid with 25 items out of 31 items. Cronbach's alpha was higher than in the original study, which ranged from 0.71 to 0.87 [11]. However, some studies did not suit 25 items, specifically the Middle East countries. Therefore, psychometric properties are critically needed in order to check instrument construct validity and reliability.

MMSS has been used in several settings and countries. Although it was developed in the United States, several other countries, including Arabic, Persian, China, Turkey, and Indonesia, have translated and validated it [12–16]. This scale aims to know the quality of nursing care from the perspective of the nurse. All translations of the scale must maintain the same level of precision as the original [17]. Therefore, accurate translation and cultural adaptation are necessary before it can be used effectively. The validity and reliability of the construct in the Indonesian version still needed to be established, even though it had been validated in other languages. This study aimed to examine the reliability and validity of the Indonesian version of the MMSS (I-MMSS).

## 2. Methods

### 2.1. Design

A cross-sectional observational prospective study was used to achieve cultural adaptation of the Indonesian version of the MMSS. This study was conducted in two phases to translate and test the construct validity and reliability of the Indonesian versions of the MMSS.

#### 2.1.1. Phase I: Cultural Adaptation and Translation of the Questionnaire

The first stage consisted of the following subphases, all of which were concerned with localizing the MMSS instrument into Indonesian [18]: (1) forward translation and compare translation between instructors, (2) back translation and compare translation between instructors, (3) compare original and translation, (4) pilot testing. Forward translator was done by a professional language translator and a professor in nursing. Then, backward translator was done by two nurses' experts from the hospital.

#### 2.1.2. Phase II: Questionnaire Test

The phase was validated through a retrospective cross-sectional observational study.

### 2.2. Participants

Three hundred-fifty Indonesian nurses were surveyed for this study. The information was collected from August to September 2022 from a random sample of inpatient nurses (IPD). The following standards were used to select participants: (1) have worked in an IPD unit for at least one year, and (2) have a bachelor's degree or diploma in nursing from an accredited university. Professionals who were not currently employed were not included in the analysis.

Selecting a sufficient sample size is a crucial decision. Regrettably, no agreed-upon criteria for validation studies exist in the existing literature [19]. The majority of them are approximations that range from three to 20 items per variable [20]. There are a total of 31 items on the MMSS scale, so the 350 participants fall within the established norms. Moreover, the research employed a convenience sampling technique to select nurses from seventeen hospitals. The questionnaires were disseminated and retrieved until the desired number of participants was achieved.

### 2.3. Data Collection Instruments

I-MMSS has 31 items with eight dimensions, including satisfaction with external awards (3 items), satisfaction with service schedules (6 items), satisfaction with the balance between family and work (3 items), satisfaction with relationships with coworkers (2 items), satisfaction with the opportunity to interact in the work environment (4 items), satisfaction with the opportunity to develop professionally (4 items), satisfaction with public recognition (4 items), and satisfaction with responsibility (5 items). The scoring and interpretation of the score is a five-point Likert scale. The score ranged from 1 = strongly disagree to 5 = strongly agree.

According to the prevailing agreement among professionals, the I-MMSS score is evaluated using a numerical continuum that spans from 1 to 5, encompassing five discrete gradations. The class interval formula *x*¯ = (*x*¯ max—*x*¯ mix)/*k* has been utilized to categorize the mean score into five levels. Furthermore, in order to avoid the incidence of intersecting intervals, a margin of 0.01 was introduced to every subsequent lower boundary, as indicated in citation [18]. The mean scores of the I-MMSS have been classified into five levels of interpretation. The aforementioned tiers are categorized as follows: the study revealed that the level of satisfaction among nurses was generally low, with a range of scores falling between 1.00 and 2.60 for poor and very poor levels of satisfaction. A fair level of satisfaction was observed within the range of 2.61 to 3.40, while a good level of satisfaction was noted between 3.41 and 4.20. The highest level of satisfaction, falling within the range of 4.21 to 5.00, was classified as very good.

### 2.4. Ethical Considerations

The research project adhered to the principles delineated in the Declaration of Helsinki, which served as the guiding document for the study. The Ethics Committee of the National Research and Innovation Agency of the Republic of Indonesia (BRIN) granted approval to the research protocol (Ref. No: 176/KE.01/SK/8/2022), and written authorization was also obtained from the hospital's managing director. Prior to affixing their signature to the document, every participant submitted a written informed consent form and furnished details pertaining to their personal information and participation in the research. The study's participants provided voluntary consent to participate in the investigation, without any form of coercion or inducement. The participants were granted unrestricted autonomy to engage in sketching activities at any juncture during the data collection process.

### 2.5. Data Collection Procedure

Upon receiving the official permission letter from the hospital director, we proceeded to establish communication with either the nursing director or the chief nursing officer. The ward coordinator or the unit coordinator ensured that the research was comprehensively elucidated to all individuals present. The questionnaires were distributed to the nurses by the coordinator of the respective ward or unit. After the research team member provided a comprehensive explanation of the study to all participants, they were instructed to complete a questionnaire promptly. The completed questionnaires were collected by the participants and placed in a secure container situated at the nursing station of their designated units. The aforementioned container was exclusively available to the individuals responsible for overseeing the administration of the research investigation.

### 2.6. Statistical Analysis

Using LISREL 8.72, this study conducted a confirmatory factor analysis (CFA) to assess the validity of the construct. Factor analysis is the go-to statistical method for examining test dimensions and subscales via score data. Experimenters can choose between an exploratory and a confirmatory factorial design. The MMSS is a theory-based test, so it makes sense to use a confirmatory analysis to see if it follows the same format as the original data collection instrument. Each of the following three steps must be completed:

The goodness-of-fit index (GFI) > 0.90, the comparative fit index (CFI) > 0.90, the standardized root mean square residual (SRMR) 0.08, and the root mean square error of approximation (RMSEA) 0.07 were used to assess the congruence of the measurement model with the research data. Items' factor loadings above 0.3 and a significant T-value greater than 1.96 constitute the cutoff [21].

Consistent with the recommendation of Hair et al., convergent validity was evaluated using the average variance extracted (AVE) statistic. When the variance extracted values are high, it means that the indicators are good surrogates for the latent variable. To meet recommendations, a construct's average variance extracted value must be greater than 0.5 [21]. If AVE is less than 0.5, but the composite reliability is higher than 0.6, the convergent validity of the construct is still adequate [22].

Both composite reliability and Cronbach's alpha were used to evaluate the reliability of the survey. All composite construction reliabilities were greater than the critical value of 0.7, indicating high reliability [21]. Most often, this will be a coefficient like Cronbach's alpha. Correlations between items and overall were also reported as means. When the former is between 0.3 and 0.7, it is considered normal, while the latter is fine once it rises above 0.3 [23].

## 3. Results

### 3.1. Translation

The translation procedure was gone through four phases. First, one member of the research team met with the original researcher, who translated the instrument, to determine whether or not the measure was culturally relevant, and this study used this meeting to assess the instrument's conceptual equivalence. Second, the translator, fluent in English and Indonesian and well-versed in the culture to which the MMSS administered, worked to translate the items from the English version of the MMSS into Indonesian and back into English. Third, the translated version was reviewed for relevance and conceptual ambiguities by a panel of experts consisting of three nurses with doctoral degrees and extensive clinical experience and one official translator. Forth, a preliminary version of the instrument was tested with native speakers of the target language. Once the questionnaire was translated into Indonesian, it underwent a series of pilot tests (*n* = 30) among a representative sample of nurses from the intended sample. This was done to ensure that the questionnaire's instructions, items, and response format were all easily understood. A dichotomous scale was used to have each participant rate the items and directions (clear or unclear). Since the pilot test revealed that there were no questions that needed clarification, the final version of the questionnaire did not require modifications.

### 3.2. Construct Validity

The initial model only had a significant result for *χ*^2^ (0.00) and CFI (0.95), and others were insignificant. After following the modification indices of error covariance on each observed variable, the criteria can be achieved, including *χ*^2^ (0.00), *χ*^2^/d*f* (2.21), CFI (1.00), GFI (0.97), AGFI (0.96), RMSEA (0.06), and SRMSR (0.06) (Table 1). The findings indicated that the I-MMSS exhibited construct validity, as evidenced by significant results of eight dimensions and 31 items.

**Figure 1:**
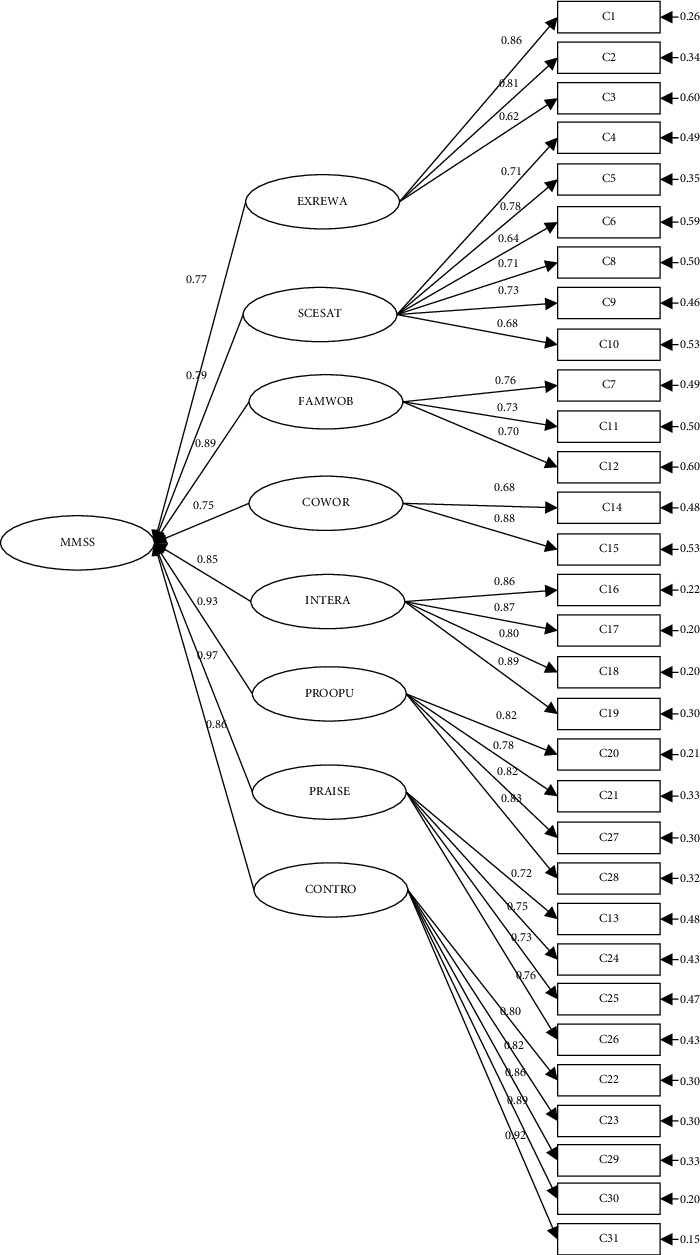
Modified measurement model.

The CFA revealed that the three subscales established in each dimension of the original MMSS were statistically significant in the Indonesian version. The loading of standardized factors of each dimension ranged from 0.75 to 0.97 at a statistically significant level of 0.05. Factor loadings for each item ranged from 0.62 to 0.86 for extrinsic, from 0.64 to 0.78 for scheduling, from 0.59 to 0.72 for family and work balance, from 0.68 to 0.88 for coworker, from 0.80 to 0.89 for interaction, from 0.72 to 0.76 for praise/recognition, from 0.78 to 0.83 for professional opportunities, and from 0.80 to 0.92 for control/responsibility. The details of unstandardized factor loading, standard error, standardized factor loadings, *R*2 and error are shown in Supplementary Appendix (available here).  Figure 1 reports the obtained standardized factor loading. Another important issue of the CFA is to check the fit of the factorial model.

### 3.3. Composite Reliability and Average Variance Extracted

The composite reliability of I- MMSS for each of its latent variables ranged from 0.70 to 0.93, with higher values indicating more robust reliability (Table 2). Composite reliability was highest for the control/responsibility dimension (*ρc* = 0.93), then for the interaction dimension (*ρc* = 0.92), and finally for the professional opportunity dimension of professional opportunities (*ρc* = 0.89). Overall, Cronbach's alpha indicated a level of trustworthiness within the data of 0.96. Cronbach's alpha for the full five-factor scale was between 0.69 and 0.93. Table 2 displays the item-to-total correlation, which varied from 0.49 to 0.79.

The average variance extracted using I-MMSS for each latent variable varied between 0.44 and 0.66 (Table 2). A moderate amount of the variance of the latent variable's variance (*ρv* = 0.44) can be explained by the dimensions of family-work balance. Others were explained at a high level.

## 4. Discussion

The CFA found that in the I-MMSS questionnaire, all eight of the subscales established in the original version's dimensions were significant. Several goodness-of-fit indices can be used, depending on the characteristics of the sample and the study, but different viewpoints and thresholds are presented in the literature [24]. There is currently no “winning” fit index that can be accepted by everyone. The study followed Hair et al. [21] which considered *χ*^2^, *χ*^2^/d*f*, CFI, GFI, AGFI, SRMR, and RMSEA.

Factor loading showed the variance explained by the variable on that factor. In the factor analysis approach, 0.3 or higher factor loading represents that the factor extracts moderate variance from that variable [25]. The latent dimension variable constructed well I-MMSS (from 0.75 to 0.97). The highest factor loading of the dimensions was praise/recognition at 0.97 (*R*^2^ = 0.94), which was followed by professional opportunities at 0.93 (*R*^2^ = 0.87) and family and work balance at 0.89 (*R*^2^ = 0.79).

The majority of items had a factor loading higher than 0.6. The highest factor loading was item 31 (*b* = 0.92) “participation in decision making,” followed by item 30 “control of the workplace environment” and then item 19 “interaction with others in healthcare” with a factor loading of 0.89 on each item. Those showing items 31 and 30 were dominantly influenced to construct dimension control/responsibility, as well as item 19 was the highest item to construct interaction. The study supported the previous psychometric study in Canada that found that control/responsibility and interaction had a significant result [26]. Additionally, former studies found that nurses feel satisfied when they have appropriate control of and responsibility for their job [27, 28].

Meanwhile, item 12 “childcare facilities on workplace” was the lowest factor loading of 0.59, which was followed by item three “benefit if resigned” and item six “clinical ladder” at 0.62 and 0.64, respectively. Those elements were the lowest to influence the dimensions to construct, and the results were above the minimum 0.3 [21]. These findings could be considered as the nurse feeling unsatisfied related to them. Most of the hospitals in Indonesia, public and private, could not provide childcare facilities. The minimum benefit for resigning is because the person will lose their insurance. In addition, the clinical ladder in some countries might not work well. A redundant role or act impacts implementation in the hospital [29].

The composite reliability and Cronbach's Alpha reliability were almost the same. It showed that I-MMSS has a strong internal consistency [30]. The highest internal consistency was control/responsibility (*ρc* = 0.93) and was followed by the dimension of interaction and the professional opportunities at *ρc* = 0.92 and *ρc* = 0.92, respectively. However, the dimension of family and work balance was the lowest internal consistency (*ρc* = 0.70), which was followed by the coworker (*ρc* = 0.76) and extrinsic (*ρc* = 0.81).

The convergent validity showed that all constructs had *ρv* > 0.5, except family and work balance (*ρv* = 0.44). However, based on Fornell and Larcker [22], if the composite reliability >0.6, it was not an issue for the convergent validity.

### 4.1. Implications for Nursing Management

This finding has potential implications for clinical practice policy. MMSS could accurately measure the nurse's feelings about their job in their workplace, which would be beneficial for the nurse manager or the nursing organization to check the nurse's condition. The dimension of MMSS provides nurse feelings about salary, working hours, childcare facilities, relation with nursing peers, social contact in workplace, opportunities to step up the career ladder, manager or leader recognition, and voice of decision making. The nurse manager and the chief nursing officer can check it regularly to maintain nurse performance. In addition, the dimensions of MMSS can be a framework for nurse organizations to promote nurse satisfaction in hospitals. The fresh graduate nurse or experienced nurse would consider moving or applying to the new workplace according to the MMSS framework. This tool can become an initial assessment of hospital policy if nurses underperform which would help the nurse problem in the unit.

### 4.2. Limitations and Future Directions

This study is the first study to investigate MMSS translation and psychometric properties in Indonesia. This finding could be as a reference for psychometric properties, specifically construct validity and reliability, as well as convergent validity. However, there are some limitations. To begin, other forms of construct validity testing, such as concurrent and discriminant validity, were not included in this investigation. Second, the study could not perform the test-retest reliability. Further studies must measure the concurrent and discriminant validity, as well as the test-retest reliability with the different settings and a larger sample size.

## 5. Conclusions

According to the results of the research, the Indonesian version of the MMSS demonstrates satisfactory levels of construct validity, convergent validity, and internal consistency. I-MMSS can be used to evaluate the perceptions of IPD nurses in terms of how they feel about salary, scheduling, family and work balance, coworker, interaction, praise/recognition, professional opportunities, and control/responsibility in the Indonesian hospital. These perceptions can be evaluated in terms of how nurses feel about professional opportunities, control, and responsibility.

## Figures and Tables

**Table 1:** Goodness-of-fit statistics of the Indonesian version of the McCloskey/Mueller Satisfaction Scale (I-MMSS) measurement model (*N* = 350).

Relative fit index	Initial model	Modified model
*χ* ^2^	0.00	0.00
*χ* ^2^/d*f*	5.98	2.21
CFI	0.95	1.00
GFI	0.68	0.97
AGFI	0.63	0.96
RMSEA	0.11	0.06
SRMSR	0.12	0.06

**Table 2:** Summary of composite reliability, extracted average variance, and internal consistency.

Latent variables	Item	Composite reliability of latent variables	Average variance extracted	Cronbach's *α*	Item to total correlation
Extrinsic	3	0.81	0.59	0.77	0.58–0.62
Scheduling	6	0.85	0.51	0.85	0.44–0.77
Family and work balance	3	0.70	0.44	0.69	0.45–0.57
Coworker	2	0.76	0.62	0.75	0.6
Interaction	4	0.92	0.73	0.93	0.77–0.88
Praise/recognition	4	0.83	0.55	0.84	0.57–0.68
Professional opportunities	4	0.89	0.66	0.89	0.71–0.81
Control/Responsibility	5	0.93	0.74	0.93	0.74–0.88
Overall	31			0.96	0.49–0.79

## Data Availability

Datasets are available from the corresponding author upon reasonable request.

## Supplementary Materials

Supplementary Materials The supplementary appendix showed unstandardized (b) and standardized (B) factor loadings, standard error (SE), *t*-value, *R* square, and error of each item. Additionally, the COSMIN checklist was used to guide the report of this study.
