# Supplementary material for: Reliability and Validity of the Indonesian Version of the McCloskey/Mueller Satisfaction Scale
Source: J Nurs Manag. 2023 Jul 13;2023:9999650. doi: 10.1155/2023/9999650 (PMC11918970; doi:10.1155/2023/9999650)
Supplement: Supplementary Materials — The supplementary appendix showed unstandardized (b) and standardized (B) factor loadings, standard error (SE), t-value, R square, and error of each item. Additionally, the COSMIN checklist was used to guide the report of this study. [file 9999650.f1.docx]

**Supplementary Appendix.** Factor Loading and Factor Score Regression of Indonesian version of the McCloskey/Mueller Satisfaction Scale (I-MMSS) (N = 350)

| McCloskey/Mueller Satisfaction Scale of latent construct | b | SE | B | t-value | R^2^ | Error |
| --- | --- | --- | --- | --- | --- | --- |
| **Extrinsic** |  | **.01** | **.77** | **45.09** | **.60** | **.40** |
| Item 1 | .94 | .10 | .86 | 3.09 | .74 | .31 |
| Item 2 | .86 | .03 | .81 | 27.25 | .66 | .39 |
| Item 3 | .72 | .03 | .62 | 23.78 | .39 | .82 |
| **Scheduling** |  | **.01** | **.79** | **45.13** | **.62** | **.38** |
| Item 4 | .72 | .08 | .71 | 6.22 | .51 | .51 |
| Item 5 | .73 | .04 | .78 | 17.20 | .64 | .34 |
| Item 6 | .71 | .04 | .64 | 17.34 | .41 | .74 |
| Item 8 | .83 | .04 | .71 | 18.99 | .50 | .67 |
| Item 9 | .85 | .04 | .73 | 19.17 | .54 | .62 |
| Item 10 | .79 | .04 | .68 | 17.67 | .47 | .72 |
| **Family and work balance** |  | **.01** | **.89** | **48.21** | **.79** | **.21** |
| Item 7 | .76 | .08 | .72 | 6.27 | .51 | .54 |
| Item 11 | .73 | .04 | .67 | 19.40 | .44 | .67 |
| Item 12 | .70 | .04 | .59 | 18.48 | .34 | .94 |
| **Co-worker** |  | **.01** | **.75** | **40.27** | **.56** | **.44** |
| Item 14 | .49 | .08 | .68 | 3.17 | .47 | .28 |
| Item 15 | .63 | .08 | .88 | 7.87 | .78 | .11 |
| **Interaction** |  | **.01** | **.85** | **44.00** | **.72** | **.28** |
| Item 16 | .66 | .08 | .86 | 1.75 | .74 | .15 |
| Item 17 | .60 | .04 | .87 | 13.41 | .76 | .11 |
| Item 18 | .54 | .04 | .80 | 13.09 | .64 | .16 |
| Item 19 | .66 | .05 | .89 | 13.97 | .79 | .12 |
| **Praise/Recognition** |  | **.01** | **.97** | **50.47** | **.94** | **.06** |
| Item 13 | .62 | .08 | .72 | 4.30 | .52 | .35 |
| Item 24 | .59 | .04 | .75 | 13.50 | .57 | .26 |
| Item 25 | .52 | .04 | .73 | 12.83 | .53 | .24 |
| Item 26 | .67 | .04 | .76 | 14.74 | .57 | .33 |
| **Professional opportunities** |  | **.01** | **.93** | **55.50** | **.87** | **.13** |
| Item 20 | .62 | .08 | .82 | 2.33 | .67 | .19 |
| Item 21 | .65 | .04 | .78 | 16.13 | .61 | .26 |
| Item 27 | .67 | .04 | .82 | 15.74 | .68 | .22 |
| Item 28 | .72 | .04 | .83 | 17.03 | .68 | .24 |
| **Control/Responsibility** |  | **.01** | **.86** | **44.86** | **.74** | **.26** |
| Item 22 | .63 | .08 | .80 | 2.73 | .64 | .22 |
| Item 23 | .62 | .05 | .82 | 13.60 | .67 | .19 |
| Item 29 | .68 | .05 | .86 | 13.87 | .73 | .17 |
| Item 30 | .70 | .05 | .89 | 13.95 | .80 | .13 |
| Item 31 | .74 | .05 | .92 | 14.45 | .85 | .09 |

The supplementary appendix showed unstandardized (b) and standardized (B) factor loadings, standard error (SE), t-value, R square, and error of each item. The unstandardized factor loading (b) of each dimension ranged from .75 to .97 at a significant statistic level of .05. The highest factor loading was praise/recognition, and the lowest was co-worker. Unstandardized factor loadings for each latent variable (b) ranged from .72 to .94 for extrinsic, from .71-.85 for scheduling, from .70 to .76 for family and work balance, from .49 to .63 for co-worker, from .54 to .66 for interaction, from .52 to .67 for praise/recognition, from .62 to .72 for professional opportunities, and from .62 to .74 for control/responsibility.
